# Management of Migraine-Associated Vestibulocochlear Disorders

**DOI:** 10.3390/audiolres13040047

**Published:** 2023-07-19

**Authors:** Kayla K. Umemoto, Karen Tawk, Najva Mazhari, Mehdi Abouzari, Hamid R. Djalilian

**Affiliations:** 1 College of Medicine, California Northstate University, Elk Grove, CA 95757, USA; 2 Division of Neurotology and Skull Base Surgery, Department of Otolaryngology-Head and Neck Surgery, University of California, Irvine, CA 92697, USA; 3 Department of Biomedical Engineering, University of California, Irvine, CA 92617, USA

**Keywords:** migraine, vestibulocochlear symptoms, lifestyle modifications, migraine prophylactic treatment

## Abstract

Migraine is a chronic neurological disorder that frequently coexists with different vestibular and cochlear symptoms (sudden hearing loss, tinnitus, otalgia, aural fullness, hyperacusis, dizziness, imbalance, and vertigo) and disorders (recurrent benign positional vertigo, persistent postural perceptual dizziness, mal de debarquement, and Menière’s disease). Despite evidence of an epidemiological association and similar pathophysiology between migraine and these vestibulocochlear disorders, patients suffering from migraine-related symptoms are usually underdiagnosed and undertreated. Current migraine treatment options have shown success in treating vestibulocochlear symptoms. Lifestyle and dietary modifications (reducing stress, restful sleep, avoiding migraine dietary triggers, and avoiding starvation and dehydration) and supplements (vitamin B2 and magnesium) offer effective first-line treatments. Treatment with migraine prophylactic medications such as tricyclic antidepressants (e.g., nortriptyline), anticonvulsants (e.g., topiramate), and calcium channel blockers (e.g., verapamil) is implemented when lifestyle and dietary modifications are not sufficient in improving a patient’s symptoms. We have included an algorithm that outlines a suggested approach for addressing these symptoms, taking into account our clinical observations. Greater recognition and understanding of migraine and its related vestibular and cochlear symptoms are needed to ensure the appropriate diagnosis and treatment of affected patients.

## 1. Introduction

Migraine headache is a complex neurological disorder that affects 1.1 billion people worldwide, making it one of the most prevalent neurological disorders [1]. It is typically characterized by moderate-to-severe episodic headaches, often accompanied by symptoms such as nausea and/or vomiting, photophobia, and phonophobia [2] Current studies have demonstrated that migraine headaches are closely associated with not only migraine-associated vertigo, a previously recognized association, but also several other vestibulocochlear disorders [3]. These disorders include vestibular migraine [4], persistent postural-perceptual dizziness [5], benign paroxysmal positional vertigo [6], Mal de Debarquement syndrome [7], sudden sensorineural hearing loss [8], tinnitus [9], hyperacusis [10], aural fullness [11], otalgia [12], and Menière’s disease [13]. It is proposed that these vestibulocochlear symptoms are manifestations of migraine in the inner ear.

Migraine-associated vestibular and cochlear disorders are often underdiagnosed and undertreated. Therefore, understanding the association between migraine and these vestibulocochlear manifestations may prove useful in developing more targeted treatments and aid in the treatment of a broader patient population previously thought to be suffering from disease processes. The purpose of this review is to discuss the different types of migraine-associated vestibular and cochlear symptoms and to provide a comprehensive overview of their pharmacological and non-pharmacological management approaches. Therefore, by offering insights into the epidemiological and pathophysiological connection between otologic symptoms and migraine, we seek to contribute to the understanding of their potential link and therapeutic approaches. Finally, this expert opinion includes an algorithm that outlines a suggested treatment strategy for addressing these cochleovestibular symptoms, taking into account our clinical observations, and highlights future directions for the treatment of these complex symptoms.

## 2. Pathogenesis

The leading theory underlying the pathophysiology of a migraine is the activation of the peripheral and central trigemino-vascular neurons, which release neuropeptides and cytokines such as CGRP, substance P, neurokinin A, and pituitary adenylate cyclase-activating polypeptide [14,15]. The abnormal release of these molecules causes vasodilation, plasma extravasation, and mast cell degranulation, generating a subsequent chronic neuroinflammatory state in the meninges [16,17,18,19,20]. Additionally, the inner ear (vestibule and cochlea) is connected via neurovascular branches to the trigeminal nervous system [21]. Therefore, the activation of the trigeminal meningeal nociceptors, inflammation of the trigeminal nerve, and vasospasm and vasodilation of the inner ear circulation cause migraine-related cochlear changes. These neurovascular changes can explain many of the symptoms seen regarding sudden hearing loss, tinnitus, aural fullness, otalgia, persistent postural-perceptual dizziness, benign paroxysmal positional vertigo, Mal de Debarquement syndrome, Menière’s disease, and some forms of vestibular migraine, thus suggesting a migraine process.

## 3. Previous and Current Management

In the past, the primary approach to treating migraine-associated vertigo was primarily symptomatic management, which included the use of anticholinergics, antihistamines, antidopaminergics, and benzodiazepines [22]. Research has been conducted to study prophylactic management for migraine-associated vertigo, including the implementation of migraine prophylactic medications, antihypertensive medications, selective serotonin reuptake inhibitors, anticonvulsants, and lifestyle management [22]; however, these studies were limited in scope and depth, partially due to the weak association made between migraine and vestibular symptoms at this time.

In recent years, more research has been conducted studying both the association between migraine and vestibulocochlear disorders and the efficacy of migraine-prophylaxis (nortriptyline, verapamil, and/or topiramate) in treating these disorders. Additionally, due to patient suspicion regarding pharmacologic treatments for reasons including cost, fear of side effects, and the belief that they lack effectiveness or lack evidence, complementary and integrative medicine alternatives are often utilized by patients to help manage migraines [23]. Examples of these pharmacological and non-pharmacological therapies are listed in Table 1. Current management techniques are focused on preventing the recurrence of these vestibulocochlear disorders, treating the symptoms, and improving the patient’s quality of life.

## 4. Migraine-Associated Vestibular and Cochlear Disorders

Since 2016, there has been increasing interest in research on several vestibulocochlear disorders and their relationships to migraine headaches. It is now believed that migraine headaches should encompass a larger group of disorders related to the inner ear, which could help clinicians to diagnose, understand, and treat these previously poorly understood disorders. A migraine can be subclassified as either a vestibular migraine or cochlear migraine, depending on the patient’s presenting symptoms. This section will further discuss the specific migraine-associated vestibular and cochlear disorders, their epidemiology, and the prior and most current treatments for these disorders.

## 5. Vestibular Migraine

Vestibular migraine (VM) is a migraine disorder that has been recognized and defined by the Bárány Society and the International Classification of Headache Disorders [25]. According to Formeister et al., VM affects 2.7% of adults in the United States [26]. The Bárány Society has defined two forms of migraine with vestibular symptoms: VM and probable VM. The exact cause of vestibular migraine is still a subject of debate, but it is believed to be a result of a central process affecting the vestibular system, which is responsible for balance. Currently, leading theories suggest that it may be triggered by neurovascular inflammation that occurs after the activation of the trigeminal neurovascular complex, which then spreads to the inner ear [27,28]. Other factors that may contribute to its development include genetic abnormalities in neuronal ion channels and the hyperactivation of certain sensory processing areas in the brain [18,27]. The presence of calcitonin gene-related peptide (CGRP) in trigeminal neurons and hair cell synapses may also play a role in pain perception and vasodilation [29,30,31]. Functional MRI studies have revealed abnormal brain activity in patients with chronic migraines, even when they are not experiencing an active migraine attack, indicating a processing-level pathology [32]. Additionally, family studies reveal autosomal dominant inheritance with incomplete penetrance, particularly in women, and a four to ten times higher prevalence compared to the general population [17,28,32]. Vestibular migraine is likely a complex, multigenic disease with varied presentations [33,34].

Definite VM is characterized by recurrent moderate-to-severe vestibular symptoms (spontaneous, positional, visually induced, or head-motion-induced vertigo or dizziness), lasting between 5 min and 72 h. At least half of these episodes should be associated with migraine headaches, photophobia and phonophobia, and/or visual aura [23,30,35]. Additionally, the Bárány Society has defined criteria for probable VM. These patients have experienced at least five episodes of moderate-to-severe vestibular symptoms that last between 5 min and 72 h, either have a history of migraines or have had an episode with migraine features, and do not have a more appropriate vestibular or International Classification of Headache Disorders diagnosis [23]. However, these criteria have been demonstrated to be too strict, and by adhering to them, clinicians only acknowledge patients with probable and definite VM [4,36], thus leaving many subtypes of VM unidentified and undertreated.

Vestibular migraine treatment is challenging, with no widespread consensus. The current treatment options are extrapolated from the non-pharmacological and pharmacological management of migraine headaches. They consist of halting an acute attack (abortive medications) or preventing future attacks (non-pharmacological treatments and prophylactic medications). Non-pharmacological treatments focus on managing migraine triggers and include changes in diet, sleep hygiene, stress management, and avoiding intense stimulation [36]. In addition, supplements such as magnesium and riboflavin were found to be helpful in preventing migraine attacks [8,37,38,39,40]. Also, vestibular rehabilitation, which involves activities aimed at enhancing spatial perception and body coordination (i.e., dancing and ping-pong), have been utilized [36]. Pharmacologic treatments have been used both to address acute VM episodes and for preventive treatment, with the exact choice of treatment depending on patient comorbidities and the medication’s side effect profile [36]. Abortive therapies include antiemetics like ondansetron, antihistamines such as meclizine, and antidopaminergics such as metoclopramide to treat nausea caused by vestibular dysfunction. In addition, triptans (almotriptan, sumatriptan, and zolmitriptan) and intravenous steroids [41,42] have been found to be effective [43,44,45]. Prophylactic medications include antiepileptic drugs such as topiramate and lamotrigine, tricyclic antidepressants such as amitriptyline, selective serotonin reuptake inhibitors (SSRIs) such as venlafaxine and paroxetine, serotonin and norepinephrine reuptake inhibitors (SNRIs), benzodiazepines, calcium channel blockers such as verapamil, and beta blockers such as propranolol [46,47,48]. CGRP monoclonal antibodies (galcanezumab) and serotonin receptor 5-HT1F agonists (lasmiditan) are novel therapies more recently approved for migraine prevention and treatment and are currently being studied regarding their ability to treat VM [36]. In 2020, the Association for Migraine Disorders identified the need for clear management recommendations for both nonpharmacologic and pharmacologic interventions as a current care gap, and an expert summit was held to encourage researchers to address these gaps [36].

## 6. Persistent Postural-Perceptual Dizziness

Persistent postural-perceptual dizziness (PPPD) is a disorder defined by long-lasting dizziness, unsteadiness, and a sensation of motion due to an inciting event (i.e., panic attacks, vestibular migraine, autonomic dysfunction, head injuries, etc.) [5]. Inciting events can cause vestibular symptoms (i.e., vestibular migraine, head injury), increase postural awareness (i.e., autonomic dysfunction), or psychological distress (i.e., panic attack) [49,50]. The current understanding of the pathophysiology of PPPD includes an inability to quickly readapt after an event that causes dizziness or vertigo, proposed to be caused by the mal-aligned processing of visual stimuli and vestibular input [51]. PPPD is associated with different comorbidities such as vestibular migraine, benign paroxysmal positional vertigo, and Menière’s disease [52]. Currently, treatment is mostly supportive, using vestibular rehabilitation therapy and cognitive behavioral therapy. Additionally, an uncontrolled study conducted by Staab et al. found that the SSRI sertraline reduced the dizziness handicap inventory scores in all patients being treated for PPPD, with 73% of patients reporting a positive response [53]. Thus, current pharmacotherapy includes selective serotonin reuptake inhibitors and serotonin-norepinephrine reuptake inhibitors [53]. However, these treatments vary in their efficacy [54].

In addition to being caused by an inciting event, some believe that the pathogenesis of PPPD may be related to migraine headaches. In fact, Bittar et al. found that 26% of patients with PPPD had migraine [55], and Staab et al. found that 16.5% of patients with PPPD had migraine [49]. Sarna et al. also recognized an increased prevalence of migraine (53%) in patients with PPPD when compared to the general population (8–13%) [5]. This association is consistent with the understanding that migraine headaches are an inciting event that triggers PPPD [5]. Since many patients with PPPD also experience migraine symptoms, Sarna et al. demonstrated that patients experienced headache relief with medication (*p* = 0.02) [5]. Therefore, if patients who present with PPPD are treated for the underlying migraine, the PPPD symptoms can also be improved [5]. More studies must be conducted on PPPD treatment with migraine prophylactic therapy.

## 7. Benign Paroxysmal Positional Vertigo

Benign paroxysmal positional vertigo (BPPV) is the most common cause of vestibular symptoms [56]. It is characterized by brief (<1 min) and recurrent episodes of vertigo or dizziness and by nystagmus triggered by lying down or turning over when supine. It is caused by the displacement of otoconia (bio-crystals of calcium carbonate) from the utricle and saccule into a semicircular canal, which disrupts normal endolymph flow and cupular deflection, resulting in positional vertigo and nystagmus [57]. BPPV is typically diagnosed using the Dix Hallpike maneuver to specify the affected canal and the pathophysiology (canalolithiasis or cupulolithiasis). The treatment of BPPV involves the use of repositioning maneuvers such as the Epley or Semont maneuvers. Though treatment with the Epley maneuver is highly effective, many patients experience recurrence, with 23–29% of patients experiencing recurrence within a year [58,59].

Understanding the etiology of BPPV is important for finding a treatment that can prevent recurrence. Several theories regarding the cause of the spontaneous utricular otolithic membrane degeneration [60] exist, including superior vestibular artery occlusion [61] and wear and tear with age, as indicated by the increase in incidence amongst the elderly population [62]. Migraine is also closely associated with BPPV. In their study, Bruss et al. showed that 50% of patients with recurrent BPPV reported migraine and the remaining 50% reported migraine-related symptoms [6]. This association is also seen in other studies. Lempert et al. found that BPPV patients are two times more likely to also have migraine headaches [63]. Kim et al. found a statistically significant association between BPPV and migraine (6.0%) when compared to a control group (2.3%) [64]. In addition to these associations, migraine headaches and BPPV often have similar clinical features regarding head motion causing vertigo, suggesting further similarities [6]. These studies suggest that recurrent BPPV may be one manifestation of migraine headaches, which further suggests that prophylactic migraine treatment may be efficacious in treating patients with recurrent BPPV [6]. Future studies will be conducted to further explore prophylactic migraine treatment in preventing the recurrence of BPPV episodes.

## 8. Mal de Debarquement Syndrome

Mal de Debarquement syndrome (MdDS), also known as disembarkment syndrome, is a condition where patients experience feelings of “rocking” as if on a boat, lasting for months to years [65]. Oftentimes, the disorder may be triggered by exposure to passive motion, such as going on a boat or plane for an extended time [66]. This is a rare disorder, which had a prevalence of 1.3% at one neuro-otology clinic [67]. It has been hypothesized that MdDS is caused by poor dampening of an oscillating multi-loop neural network between the vestibular nuclei and flocculonodular lobe [68]. Since it is still poorly understood, MdDS is managed supportively with physical therapy; some studies have tried using vestibular therapy, such as computer-assisted rehabilitation [69]. In one study, 89% of patients who were re-exposed to the passive motion (i.e., riding in a car or boat) reported temporary relief of symptoms [65].

Studies have also indicated that the incidence of migraine headaches may be 23–38% [65] or 41–46% [70] within the MdDS population, which is significantly higher when compared to the general population. This suggests that management with migraine prophylaxis may have an effect on patients suffering from MdDS. In one study, Ghavami et al. demonstrated that of the patients with MdDS who managed the condition with migraine prophylaxis (nortriptyline, verapamil, and/or topiramate), 73% experienced improvements in their symptoms [7]. Analysis showed statistically significant improvements in mental (*p* = 0.003), physical (*p* < 0.001), and social (*p* < 0.001) wellbeing [7].

## 9. Sudden Sensorineural Hearing Loss

Sudden sensorineural hearing loss (SSNHL) is characterized by the loss of at least 30 dB for three or more contiguous audiometric frequencies over a 72 h period [71]. SSNHL affects between five and twenty-seven of every hundred thousand people in the United States [72]. One review found that about 71% of SSNHL diagnoses are idiopathic, 12.8% are due to an infectious disease, 4.7% are due to an otologic disease, 4.2% are due to trauma, 2.8% are attributed to vascular or hematologic causes, and 2.3% are attributed to neoplastic processes [73]. If the cause of SSNHL is identified, then the appropriate treatment can be started. However, for the vast majority of patients presenting with no identifiable cause, management starts with first conducting audiometric testing to determine if the hearing loss is conductive or sensorineural, with the goal of starting treatment within 2 weeks of symptom onset [74]. In addition, magnetic resonance imaging or auditory brainstem response testing should be completed to test for retro-cochlear pathology [74]. After SSNHL is established, patients should be referred to a physician who can provide the appropriate treatment [74].

In addition to finding treatments for SSNHL, studies are being conducted to determine the etiology of the majority of patients presenting with SSNHL with no identifiable cause. Some studies have shown an association between SSNHL and the trigeminal innervation of the cochlea vasculature, [21,73] which is similar to the pathogenesis of migraine headaches. Additionally, Kim et al. showed that out of 44,714 migraine patients, 0.9% developed SSNHL, while out of 179,287 control patients, 0.6% developed SSNHL, presenting a statistically significant difference (*p* < 0.001) [75]. Chu et al. showed similar results, with 81.6 per 100,000 persons with migraine developing SSNHL compared to 45.7 per 100,000 persons without migraine, which suggested an incidence ratio of 1.8 [76]. Due to the association found between SSNHL and migraine headaches, Abouzari et al. suggested that when SSNHL is not associated with a structural disorder of the ear, one etiology is migraine, and thus, migraine prophylactic treatment may be efficacious [8,77]. They found that treatment of SSNHL patients with migraine prophylactic drugs (topiramate and nortriptyline) over a six-week period, in addition to oral and intratympanic steroid therapy, improved low-frequency hearing by, on average, 20 dB. Adjuvant migraine medication improved the pure tone average (PTA) from 74 ± 22 dB to 55 ± 22 dB (*p* < 0.001), which was a statistically significant improvement compared to the control group (*p* = 0.01), in addition to requiring fewer intratympanic injections (*p* = 0.04). The same group from the University of California, Irvine [78], then looked at patients with long-term sudden hearing loss (more than 3 months from onset) to see if treatment using migraine prophylactic led to an improvement in hearing. In this study, a cohort who had experienced a median of 4 months of SSNHL was treated with prophylactic migraine and intratympanic steroid therapy. The results demonstrated that 40% of patients showed improvements in speech recognition threshold (SRT), 29% showed improvements in hearing frequencies (250 and 8000 Hz), and 68% showed improvements in word recognition score (*p* < 0.001) in [78]. Analysis found statistically significant improvements in SRT (*p* < 0.01) and hearing frequencies (*p* = 0.01 at 500 Hz; *p* = 0.03 at 1000 Hz; *p* = 0.01 at low-frequency pure-tone average; *p* = 0.02 at speech-frequency pure-tone average) [78]. This further supports SSNHL being a migraine phenomenon and that migraine prophylaxis can improve long-term SSNHL.

Currently, treatment for SSNHL has been shown to be most efficacious if provided within 2 to 4 weeks of onset. There is less literature discussing treatment for after one month from the onset and for long-term SSNHL. Data from Goshtasbi et al. showed that the presence of active migraine symptoms (headaches, aural pressure) was a good prognostic indicator for SSNHL that could be treated with migraine prophylactic medications [78].

## 10. Tinnitus

Tinnitus is the perception of sounds with no external acoustic stimulus. It is a subjective phenomenon that can be described as ringing, buzzing, hissing, or other similar sounds. Tinnitus is common, with approximately 50 million adults in the United States reporting at least one episode between 1999 and 2004, and 16 million adults reporting frequent episodes [79]. Previous discussions on the pathogenesis of tinnitus have resulted in no single theory, likely due to the presence of multiple etiologies. Tinnitus involves the disruption of auditory and somatosensory input [80,81]. The dorsal cochlear nucleus (DCN) receives direct auditory input from the vestibulocochlear nerve and indirect somatosensory input from the trigeminal nerve [80,81]. Animal studies have shown that somatosensory signals from the spinal trigeminal nucleus cancel out self-produced sounds in the DCN, suggesting that this plays a role in the spontaneous nature of tinnitus [80,81]. The pathophysiology of tinnitus can be categorized into cellular, system, and other factors [80,81]. At the cellular level, tinnitus is associated with increased neural synchrony, changes in neurotransmission, and maladaptive plasticity [80,81]. The DCN acts as a tinnitus generator, while the inferior colliculus, medial geniculate body, and auditory cortex contribute to tinnitus generation at the system level [80,81]. Non-auditory structures like the parahippocampus, dorsal anterior cingulate cortex, ventral prefrontal cortex, insula, orbitofrontal cortex, posterior cingulate cortex, and precuneus are involved and exhibit altered activity and connectivity in tinnitus patients [80,81]. The interconnectedness within these structures plays a role in the perception and generation of tinnitus [80,81]. Overall, tinnitus involves disruptions in neural synchrony, neurotransmission, and the complex interplay of auditory and non-auditory structures.

Most treatment is focused on symptomatic improvement, decreasing tinnitus intensity and annoyance to the patient [82]. For treatment, most people achieve remission through natural habituation [83]. Other forms of therapy include cognitive behavioral therapy, music or sound therapy, tinnitus retraining therapy, stretching or massaging, and electrical suppression [82,84]. More current treatments include brain-based treatment approaches [85]. Pharmacotherapy that showed promising results in trials included tricyclic antidepressants and benzodiazepines [84,86]. Lidocaine also proved efficacious in treating tinnitus; however, since it can only be administered intravenously, has a short half-life, and has an adverse side effect profile, it cannot be used clinically [87]. Many trials testing various pharmacotherapies showed no efficacy when compared to placebos, as detailed in Table 2 [81,87].

Notable theories describing the pathogenesis of tinnitus include cortical theory and the dorsal cochlear nucleus theory [9,82]. Since the dorsal cochlear nucleus receives input from the vestibulocochlear nerve in addition to indirect input from the trigeminal nerve, tinnitus may be a result of damage to both cranial nerves [9]. This damage to the trigeminal nerve is also described in the pathogenesis of migraine headaches, suggesting that a subset of patients with tinnitus may be suffering from migraine with cochlear symptoms. In addition to the described pathogenesis, in a study analyzing the National Health and Nutrition Examination Survey, of 12,962 patients suffering from tinnitus, 36.6% also reported having migraine headaches. This result was statistically significant (*p* < 0.001) [9,88]. Based on this association, migraine treatments may be used to treat tinnitus. Currently, studies are being conducted to determine the efficacy of migraine prophylaxis in treating migraine-associated tinnitus.

## 11. Hyperacusis

There are several varieties of hyperacusis that have been described in the literature: loudness, annoyance, fear, and pain. Loudness hyperacusis refers to normal sounds being perceived as too loud or uncomfortable, whereas annoyance hyperacusis refers to sounds causing mood disturbances and irritation. Fear hyperacusis is characterized by increased sensitivity to loud sounds and associated avoidance behavior. Finally, pain hyperacusis is where sounds induce pain and discomfort, even at a much lower level than they would for listeners with normal hearing [92]. Some studies indicate that 8.6–15.2% of the population report experiencing symptoms of hyperacusis [93]. Little research has been conducted on understanding the mechanism behind hyperacusis; however, the current general consensus is that there is an increase in neural activity or central gain in the central auditory system, resulting in a suprathreshold intensity [94]. Most therapies for hyperacusis are symptomatic and include sound therapy, which aims to either desensitize or recalibrate the patient’s perception of sound, and cognitive behavioral therapy [94].

An association of hyperacusis has been made with chronic migraine headaches, termed phonophobia [95]; in addition, 16% of patients with hyperacusis reported having migraine headaches and the majority experienced migraine-related symptoms [10]. Thus, a study was conducted using migraine prophylactic therapy and showed a statistically significant (*p* < 0.001) improvement in hyperacusis patient’s loudness discomfort level (81.3 ± 3.2 dB to 86.4 ± 2.6 dB), with 88% reporting subjective symptomatic resolution [10]. These results indicate that there may be a migraine process occurring in patients experiencing hyperacusis and that migraine prophylactic therapy may be efficacious.

## 12. Aural Fullness

Aural fullness is a feeling of pressure or clogging in the ear. Roughly 1.4% of otolaryngology patients present with aural fullness [96]. Most cases are caused by eustachian tube dysfunction (ETD), in which case, patients are treated for the underlying cause [96]. The next most common group present with idiopathic aural fullness [96]. After other causes (e.g., tumor, third window syndrome, ETD, etc.) have been ruled out, patients with prolonged aural pressure who do not experience relief from using the Valsalva maneuver are likely suffering from a migraine-related phenomenon [11]. To better understand patients with prolonged idiopathic aural fullness, Moshtaghi et al. explored its relationship to migraine headaches. This study found that 54% of patients presenting with prolonged aural fullness also met the International Headache Society’s criteria for migraine headaches, which may indicate a relationship between the two diseases [11]. Based on this association, a trial of migraine prophylactic therapy was administered to patients with prolonged idiopathic aural fullness, and 73% of patients showed statistically significant (*p* < 0.001) improvements in the visual analog scale, suggesting that this may be a viable treatment option for these patients [11]. Additionally, Risbud et al. found that 26% of patients with aural fullness met four out of the five criteria for migraine, 48% met three out of the five criteria, and there were minimal differences in five of the twenty migraine features; thus, the researchers noted that these patients may also benefit from migraine prophylaxis [97].

## 13. Menière’s Disease

Menière’s disease (MD) is a clinical syndrome characterized by a symptomatic triad of episodic vertigo, fluctuating hearing loss, and aural symptoms such as aural fullness and tinnitus [98]. In 2015, the Bárány Society established two varieties of MD, definite and probable, which are accepted by the Equilibrium Committee of the American Academy of Otolaryngology—Head and Neck Surgery. The criteria for definite MD include the presence of two or more of the following criteria: (1) spontaneous vertigo episodes that last from 20 min to 12 h; (2) low-to-medium-frequency sensorineural hearing loss in one ear confirmed by audiometric evidence and that occurred before, during, or after the vertigo episodes; (3) fluctuating aural symptoms in the affected ear, such as changes in hearing, tinnitus, or fullness, and the absence of another vestibular diagnosis that can better explain these symptoms [99]. Probable MD has the same criteria except that the episodes of vertigo or dizziness may last between 20 min and 24 h [99].

The pathophysiology of MD is not fully understood; thus, medical professionals do not agree on one algorithm for treating MD patients. In 2016, due to the heterogeneous clinical presentation of MD, Frejo et al. created five distinct subtypes of patients diagnosed with unilateral MD, and a year later, of patients diagnosed with bilateral MD, in an effort to improve treatment choices [100,101,102]. Crossley et al. replicated these results in 2020 [103].

Current MD management is primarily symptomatic and includes dietary changes (i.e., salt restriction, reduced caffeine and alcohol consumption), stress reduction, and pharmacotherapy (i.e., hydrochlorothiazide, acetazolamide, chlorthalidone, betahistine, triamterene, short-term oral prednisone, and benzodiazepines) [104]. Vestibular rehabilitation and cognitive behavioral therapy have also been shown to be safe and effective, in addition to the Meniett system (micro-pressure pulses) [105]. Additionally, one should address any treatable underlying etiologies of migraine, including obstructive sleep apnea, at presentation [105]. Slightly more invasive procedures, including intratympanic corticosteroid injections (i.e., dexamethasone or methylprednisolone), are used after conservative measures have been tried [105]. More invasive procedures for patients with debilitating symptoms and those for whom first-line therapies have failed include endolymphatic sac shunt surgery; however, the efficacy of such procedures is unclear [104,105]. Intratympanic injections of gentamicin have also been implicated, but there is a risk of hearing loss that has reduced its favor [105]. Finally, very invasive procedures include labyrinthectomy and vestibular neurectomy, which have been very efficacious in controlling intractable vertigo but are rarely used since patients lose hearing function [105].

One current theory that attempts to understand the pathogenesis of MD is that there are mechanical and chemical changes in the cochlea due to vascular permeability changes from trigeminal sensory nerve activation [106,107]. The inner ears of patients with MD are in a chronically hydropic state [13]. It is hypothesized that when experiencing a migraine attack, the hydropic state results in an inability to regulate the cochlear vasculature against these changes [108]. This then leads to the release of substance P, neurogenic inflammation, and changes in blood flow, ultimately manifesting as MD [108]. Even when MD was initially coined by Menière in the mid-1800s, he noted an observed association between the classic symptomatic triad of MD and migraine headaches [109]. A recent study showed that 51% of MD patients experienced migraine headaches, while only 12% of the general population had migraine headaches [13]. Based on this understanding, patients with MD may be treated with migraine prophylactic therapy. Recent studies by Ghavami et al. have demonstrated that 92% of MD patients experienced a statistically significant (*p* = 0.02) improvement in quality of life after treatment with migraine prophylaxis [110].

## 14. Summary of Management

**Figure 1:**
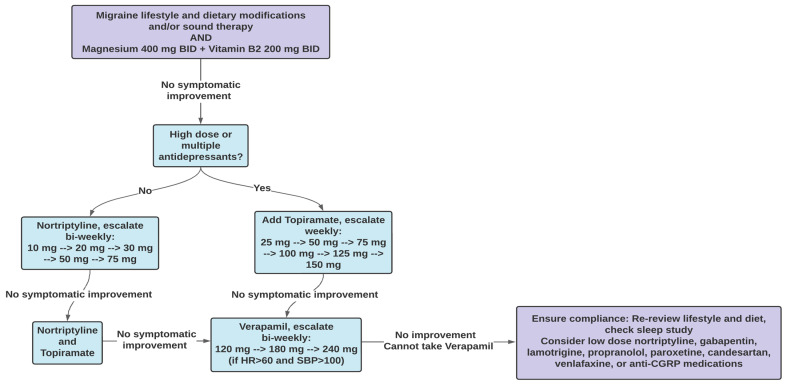
Proposed algorithm for treating patients with migraine-associated vestibular and cochlear disorders with migraine prophylactic management.

With the increased evidence of the association between migraine and various otologic symptoms, an effective approach would include migraine treatment as well as recommendations for migraine prophylaxis. According to recommendations by the American Headache Society, migraine prophylaxis management begins with education about proper lifestyle modifications; however, if these measures are insufficient, pharmacological treatments may be used [111]. A proposed algorithmic approach to migraine prophylactic management is outlined in Figure 1.

## 15. Lifestyle and Dietary Modifications and Supplements

First, patients must optimize changes in lifestyle to prevent migraines. The etiology of migraines consists of five triggers, which include: (1) stress: both psychological and physical; (2) hormonal changes: menstrual cycle, menopause, hormone replacement therapy, oral contraceptives, and testosterone supplementation in men; (3) sleep changes: too much or too little sleep, interrupted sleep, shifting sleep schedules; (4) diet: skipping meals, certain foods, dehydration; (5) intense stimulations: intense lights, sounds, smells, motion, visual motion, weather/atmospheric pressure changes (low pressure), ambient heat, cold air stimulation to the face or ear, and painful stimuli of the head and neck. Therefore, patients must be educated about and avoid these triggers to prevent migraine headache development. Migraine diaries are helpful for identifying individual triggers. In addition to lifestyle management, patients should be started on two supplements: (1) Vitamin B2 (Riboflavin) at 200 mg twice a day and (2) magnesium oxide at 400 mg twice a day [112,113,114]. More detailed recommendations can be found in Table 3 and a more detailed diet can be found in Table 4.

## 16. Pharmacological Management

If patients do not respond within six to eight weeks of diet and lifestyle changes, patients may be started on pharmacotherapy. Pharmacotherapy acts to increase the threshold required to trigger a migraine and to control underlying comorbidities. It generally takes six to eight weeks for pharmacotherapy to begin taking effect, resulting in improvements in migraine intensity and frequency [111]. If patients are not on any antidepressants, consideration should be given to recommending nortriptyline, starting at a dose of 10 mg daily and increasing bi-weekly to 20 mg, 30 mg, 50 mg, and 75 mg [9] if no cardiac arrhythmias are present. If there is still no improvement, patients should be started on topiramate, starting at a dose of 25 mg and increasing weekly to 50 mg, 75 mg, 100 mg, 125 mg, and 150 mg. If patients continue to experience no improvement and their heart rate is above 60 and systolic blood pressure is above 100, patients can then be recommended verapamil, starting at a dose of 120 mg daily and increasing bi-weekly to 180 mg and 240 mg [7,118,119,120,121]. Finally, if there is still no improvement, it is important to review the patient’s lifestyle, ensuring that they remain compliant with the recommendations, in addition to being started on low-dose nortriptyline, gabapentin, lamotrigine, propranolol, paroxetine, candesartan, venlafaxine, or anti-CGRP medications (Figure 1).

During an acute migraine episode, oral steroids, ondansetron, and/or gepants may be used as abortive therapy. It is important to note that patients may be sensitive to migraine prophylactic medications; thus, (1) drug dosage should be increased gradually until a therapeutic dose specific to the patient is found, and (2) drug combinations are often needed for substantial symptomatic relief [7]. The choice of pharmacotherapy depends on patient comorbidities and their ability to tolerate side effects.

## 17. Future Directions

Due to the association found between migraine headaches and vestibulocochlear symptoms, studies are currently being conducted to further investigate the efficacy of migraine prophylactic treatments on patients with migraine-associated vestibular and cochlear disorders. These studies will isolate lifestyle management and migraine prophylactic treatments to further refine the treatment regimen. Additionally, adjustments in the International Classification of Headache Disorders criteria for migraines should encompass vestibulocochlear symptoms, and providers should be educated about these adjustments [33].

## 18. Conclusions

There is increasing evidence that migraine headaches are closely associated with migraine-associated vestibulocochlear disorders. This association has important implications for the prospect of developing more targeted therapies for previously difficult-to-treat vestibulocochlear symptoms. Migraine prophylactic therapies, which include lifestyle management and pharmacotherapy, have shown efficacy in treating migraine-associated vestibulocochlear disorders.

## Figures and Tables

**Table 1:** Previously reported management techniques for migraine-associated vertigo [24].

Management	Examples	Abortive	Prevention
Abortive/symptomatic management: vestibular suppressants	Anticholinergics (scopolamine, homatropine) Antihistamines (meclizine, diphenhydramine) Anti-dopaminergic (prochlorperazine, metoclopramide) Benzodiazepines	✔	✔
Initial prophylactic pharmacologic recommendations	Low-dose nortriptyline Beta blocker (propranolol) Referral to a neurologist		✔
Neurologist-recommended prophylactic medications	First-line treatments Valproic acid Topiramate Some Efficacy Selective serotonin reuptake inhibitors/serotonin-norepinephrine reuptake inhibitors (fluoxetine, venlafaxine) Little Efficacy Carbamazepine Gabapentin Calcium channel blockers (verapamil)		✔
Lifestyle Management	Diet Physical therapy Acupuncture		✔
Complementary and Integrative Medicine Alternatives for Tinnitus	Cognitive behavioral therapy Yoga Neurofeedback Hypnosis Acupuncture Supplements Zinc Vitamin B2 Antioxidants Melatonin		✔
Complementary and Integrative Medicine Alternatives for Vertigo	Yoga Physical therapy Acupuncture Supplements Ginkgo biloba with/without neurofeedback Zinc		✔

**Table 2:** Studied medications for the treatment of tinnitus [81,87,88,89,90,91].

Medication	Dosage	Results
Lidocaine	1–2 mg/kg of body weight IV for 3–4 min	Complete or partial suppression
Amitriptyline	50–100 mg/day for 6 weeks	Decreased intensity and subjective relief
Nortriptyline	50–150 mg/day for 6 weeks	Decreased loudness
Sertraline	25–50 mg/day for 16 weeks	Decreased loudness and severity
Gabapentin	2400 mg/day for 20 weeks ***	Decreased annoyance
Alprazolam	0.25–0.5 mg/day for 1 week. Max 1 mg/day ***	Decreased loudness
Clonazepam	Not applicable	Decreased annoyance and intensity
Acamprosate	333 mg TID for 3 months ***	Improvements
Neramexane	25–75 mg/day for 16 weeks	Decreased annoyance
Betahistine	48 mg/day for 3 months	Slight improvement in loudness and tinnitus handicap inventory
Cilostazol	200 mg/day for 4 weeks	Decreased Visual Analogue Scale score
Melatonin	3 mg/day for 30 days	Decreased tinnitus intensity in patients with insomnia
Misoprostol	Increasing dosage for 4 months	Decreased loudness
Ondansetron	16 mg/day for 4 weeks	Improvements in tinnitus severity index score
Oxytocin	16 IU single dose	Decreased Clinical Global Impressions score
Pramipexole	Max dose of 0.7 mg TID for 4 weeks	Decreased annoyance
Vitamin B12	2500μg weekly for 6 weeks ***	Improvements in patients with vitamin B12 deficiency
Paroxetine, Trimipramine, Carbamazepine, Lamotrigine, Baclofen, Diazepam, Memantine, Cyclandelate, G. biloba, Piribedil, Vardenafil		No significant difference

*** No significant difference at lower doses and with shorter duration of treatment.

**Table 3:** Recommendations for avoiding physiologic triggers [1,115,116].

Physiologic Trigger	Recommendations
Sleep *	- Find and maintain a consistent sleep schedule for all days of the week - Use guided meditation prior to sleep - Avoid looking at screens and reduce lights one hour prior to sleep - Treat other sleep conditions before treating the migraine (e.g., sleep apnea, insomnia)
Diet *	- Maintain a strict eating schedule - Eat when hungry - Drink ≥70 oz of water per day, more with exercise or when outdoors in the heat - Follow the migraine diet
Stress *	- Exercise, starting with 5 min, gradual 1–2 min/week increase, with a goal of 20–30 min, three to five times a week. A stationary bike seems to be tolerated for patients with dizziness - Practice meditation

* Level of Evidence 1.

**Table 4:** Recommended Migraine Diet [115,117].

Category	Avoid, Reduce, or Limit	Acceptable Foods
Caffeine *	Coffee, tea Caffeinated soda	Decaffeinated tea Herbal tea Caffeine-free soda Fruit juice (non-citrus)
Snacks *	Chocolate Nuts Dry fruit (raisins, etc.)	Seeds Sherbet, ice cream Cakes
Alcohol *	Fermented alcohol (e.g., wine, beer, etc.)	Non-alcoholic beverages Highly distilled alcohol (e.g., vodka)
Dairy *	Most cheeses: brie, boursault, blue, cheddar, brick, camembert, mozzarella, romano, emmental, gouda, parmesan, provolone, swiss, roquefort, stilton Sour cream	Other cheeses (American, cream cheese, Velveeta, cottage, farmer, ricotta) Milk, rice milk Oatmeal Egg substitutes, egg yolk
Cereals and Grains *	Fresh bread, bagels, donuts, yeast products Yeast extracts, sourdough, brewer’s yeast	Commercial breads (white, wheat, multi, rye, Italian) English muffins Crackers, potatoes, noodles, toast, rice, spaghetti
Meats *	Aged, canned, cured, pickled, salted, and dried meats Processed meats (pepperoni, hot dogs, bologna, salami, pre-packaged deli meats, jerky, sausages) Any processed protein that contains tyramine	Fresh/unprocessed meats, fish, poultry, veal, lamb, tuna
MSG * (Monosodium Glutamate)	Soy sauce, bouillon cubes, canned soups, tenderizers, meat, seasoned salts Foods containing “hydrolyzed protein products” or “autolyzed yeast”, “autolyzed protein”, “hydrolyzed yeast” Pickled, preserved, or marinated foods Asian food sauces Off-the-shelf salad dressings	Salt and other spices Butter, margarin White vinegar
Sweetener *	Aspartame (Equal, Nutrasweet)	Sucrose (sugar), high-fructose corn syrup, sucralose, saccharin
Vegetables *	Pole or broad beans, Italian beans, lima beans, snow peas, lentils, navy beans, fava beans, pea pods, pinto beans, garbanzo beans Sauerkraut, pickles, kimchi (tyramine) Onions, olives	Asparagus, broccoli, garlic, beets, carrots, pumpkins, squash, lettuce, spinach, string beans, tomatoes All not listed
Fruit *	Avocados, papaya, figs, passion fruit Bananas and citrus fruit (orange, grapefruit, lime, lemon) Overly ripened fruits (high in tyramine)	Apples, berries, hard peaches, hard pears

* Level of Evidence 1.

## Data Availability

No new data were created or analyzed in this study. Data sharing is not applicable to this article.
